# World Health Organisation Disability Assessment Schedule (WHODAS 2.0): development and validation of the Nigerian Igbo version in patients with chronic low back pain

**DOI:** 10.1186/s12891-020-03763-8

**Published:** 2020-11-17

**Authors:** Chinonso Nwamaka Igwesi-Chidobe, Sheila Kitchen, Isaac Olubunmi Sorinola, Emma Louise Godfrey

**Affiliations:** 1 Department of Medical Rehabilitation, Faculty of Health Sciences and Technology, College of Medicine, University of Nigeria, Enugu Campus, Nigeria; 2 Department of Physiotherapy, School of Population Health Sciences, Faculty of Life Sciences and Medicine, King’s College London, London, UK; 3 Department of Psychology, Institute of Psychiatry, Psychology and Neuroscience, King’s College London, London, UK

**Keywords:** Disability, Cross-cultural, Psychometric, Igbo World Health Organisation disability assessment schedule, Africa, Nigeria, Rural, Low back pain

## Abstract

**Background:**

Globally, the leading cause of years lived with disability is low back pain (LBP). Chronic low back pain (CLBP) is responsible for most of the cost and disability associated with LBP. This is more devastating in low income countries, particularly in rural Nigeria with one of the greatest global burdens of LBP. No Igbo back pain specific measure captures remunerative or non-remunerative work outcomes. Disability measurement using these tools may not fully explain work-related disability and community participation, a limitation not evident in the World Health Organisation Disability Assessment Schedule (WHODAS 2.0). This study aimed to cross-culturally adapt the WHODAS 2.0 and validate it in rural and urban Nigerian populations with CLBP.

**Methods:**

Translation, cultural adaptation, test–retest, and cross-sectional psychometric testing was performed. WHODAS 2.0 was forward and back translated by clinical/non-clinical translators. Expert review committee evaluated the translations. Twelve people with CLBP in a rural Nigerian community piloted/pre-tested the questionnaire. Cronbach’s alpha assessing internal consistency; intraclass correlation coefficient and Bland–Altman plots assessing test–retest reliability; and minimal detectable change were investigated in a convenience sample of 50 adults with CLBP in rural and urban Nigeria. Construct validity was examined using Spearman’s correlation analyses with the back-performance scale, Igbo Roland Morris Disability Questionnaire and eleven-point box scale; and exploratory factor analysis in a random sample of 200 adults with CLBP in rural Nigeria. Ceiling and floor effects were investigated in both samples.

**Results:**

Patient instructions were also translated. ‘Waist pain/lower back pain’ was added to ‘illness(es)’ to make the measure relevant for this study whilst allowing for future studies involving other conditions. The Igbo phrase for ‘family and friends’ was used to better represent ‘people close to you’ in item D4.3. The Igbo-WHODAS had good internal consistency (α = 0.75–0.97); intra class correlation coefficients (ICC = 0.81–0.93); standard error of measurements (5.05–11.10) and minimal detectable change (13.99–30.77). Igbo-WHODAS correlated moderately with performance-based disability, self-reported back pain-specific disability and pain intensity, with a seven-factor structure and no floor and ceiling effects.

**Conclusions:**

Igbo-WHODAS appears psychometrically sound. Its research and clinical utility require further testing.

**Supplementary Information:**

The online version contains supplementary material available at 10.1186/s12891-020-03763-8.

## Introduction

Measures for low back pain (LBP) disability are mostly self-reported due to their low cost and ease of administration including reduced patient burden and non-invasiveness. Moreover, assessment of disability through self-report may be comparable to objective disability measurements [1] and is sometimes more reliable than objective assessments. Disability constructs such as participation restriction, may be more directly measured subjectively through self-reports. In contrast, performance-based disability measures may be impairment focused overlooking other important dimensions of disability such as activity limitations and participation restrictions [2]. This is misleading as people with impairment may not experience disability, or do so at varying levels depending on personal, physical and social barriers/facilitators in different contexts.

There are several back pain specific self-report measures, the most commonly used being the Roland-Morris Disability Questionnaire and the Oswestry Low Back Pain Disability Questionnaire. None of these measures cover remunerative or non-remunerative work outcomes, which is an aspect of participation [3, 4]. This implies that disability measurement using these tools may not fully explain work-related disability, community participation, and other domains of participation which are likely to be context-specific. This limitation is not evident in the World Health Organisation Disability Assessment Schedule (WHODAS 2.0), an international classification of functioning, disability and health (ICF) based generic disability measure. The measure has distinct activity and participation domains, that include work-related disability and community participation [5]. Therefore, the WHODAS 2.0 might be one of the best measures for assessing LBP disability as it reflects the biopsychosocial model of disability. The WHODAS 2.0 has been translated into 47 languages; used in 94 countries; and employed in 27 research areas [6]; and has a Nigerian Yoruba version [7].

However, the original WHODAS 2.0 is in English, making it difficult to use in clinical and epidemiological studies involving low literate non-English speaking people in rural and urban Nigeria. This is particularly important as Nigeria (particularly rural Nigeria) has one of the greatest burdens of LBP globally. Therefore, this study aims to translate, culturally adapt and investigate the validity and reliability of the Igbo version of the WHODAS 2.0 in rural and urban populations in Nigeria.

## Methods

This study was conducted in line with the COnsensus-based Standards for the selection of health Measurement INstruments (COSMIN) Study Design checklist for Patient-reported outcome measurement instruments [8].

### Ethical concerns

King’s College London (Ref: BDM/13/14–99) and University of Nigeria Teaching Hospital (Ref: UNTH/CSA/329/Vol.5) gave ethical approval. The World Health Organisation gave permission to adapt the measure. Interested participants signed or thumb printed on the consent forms following a detailed verbal and written explanation of the study, and after being given 3 days to decide whether to participate in the study.

### Study designs

This study involved cross-cultural adaptation, test-retest measurements and cross-sectional study of psychometric properties of the Igbo version of the WHODAS 2.0.

### Outcome measurement tools

#### World Health Organisation disability assessment schedule (WHODAS 2.0)

The WHODAS 2.0 is a comprehensive measure that assesses disability within the ICF biopsychosocial model of disability. It emphasizes the six domains of cognition, mobility, self-care, getting along with people, life activities and participation – including work-related disability. The cognition domain measures an individual’s difficulty in understanding and communicating. The mobility domain quantifies a person’s difficulties in getting around. The self-care domain assesses someone’s difficulties in taking care of oneself. The getting along with people domain measures an individual’s difficulties in getting along with people. The life activities domain assesses the difficulty with which activities involved in maintaining an individual’s household or work/school are performed. The participation domain measures a person’s difficulty with participating in their society and the impact of the specific health problem on them and their family. These difficulties are measured within the last 30 days. The measure has good face and content validity, construct validity, internal consistency, test-retest reliability and responsiveness. The 36-item interviewer-administered version (with simple and complex scoring methods) was used due to its relevance in populations with low literacy. Simple scoring involves assigning values “none” =1, “mild” =2 “moderate” =3, “severe” =4 and “extreme” =5, which are simply added up without weighting of individual items. However, this method may not be comparable across populations and conditions. Therefore, the complex scoring method was used in this study. Complex scoring is an “item-response-theory” (IRT) based scoring that takes into consideration multiple levels of difficulty for each item. It involves summing recoded item scores in each domain, summing all six domain scores, and converting the summary score into a metric ranging from 0 (no disability) to 100 (full disability) [5].

### Cross-cultural adaptation process

Translation is the linguistic paraphrasing of a questionnaire. Conversely, cross-cultural adaptation involves translation and cultural adaptation to enable the content validity of the instrument to be at similar conceptual levels in different contexts [2].

#### Participants involved in the cross-cultural adaptation process

One clinical physiotherapist who had practised for 16 years in Nigeria and three non-clinical translators (one native English speaker [bilingual in English and Igbo], one native Igbo speaker [bilingual in Igbo and English], and one English/Igbo linguistic expert) were the translators. An English health psychologist with expertise in research methodology and an English academic physiotherapist working in the United Kingdom, an Igbo clinical psychologist and an Igbo clinical physiotherapist working in Nigeria, made up an external expert review committee. A convenience sample of 12 adults living with non-specific CLBP in rural Nigeria who had participated in a previous study [9] and gave informed consent, were involved in piloting/pre-testing the adapted measure (qualitative assessment of content validity).

#### Procedure adopted for cross-cultural adaptation

The original WHODAS 2.0 was translated and culturally adapted using evidence-based guidelines [8, 10, 11] as illustrated in Table 1 below.

**Table 1:** Process of cross-cultural adaptation

**Step 1:** Two forward translations of the original WHODAS 2.0 to Igbo	
A. T1 (Igbo) version: bilingual Physiotherapist (native Igbo speaker, bilingual in Igbo and English)	
B. T2 (Igbo) version: bilingual non-clinical translator (native Igbo speaker, bilingual in Igbo and English)	
**Stage 2:** Synthesis of the two forward translations (T1 & T2) by the two forward translators, with CNI-C mediating discussion, to produce T-12 (Igbo) version.	
**Stage 3:** Two back translations of T-12 (Igbo) version to English	
i. BT1 (English) version: non-clinical translator (English/Igbo linguistic expert)	
ii. BT2 (English) version: non-clinical translator (native English speaker, bilingual In English and Igbo)	
iii. CNI-C: reviewed and summarised differences in BT1 and BT2 versions	
**Stage 4**: Expert and translation committee review produced pre-final Igbo WHODAS 2.0. CNI-C mediated discussion of translations and discrepancies in T1, T2, T-12, BT1 and BT2 versions with translators and experts in UK and Nigeria.	
**Stage 5**: CNI-C piloting/pre-testing the pre-final Igbo WHODAS 2.0 with patients to produce the final Igbo-WHODAS 2.0.	
***CNI-C: The first author***	

First step – the WHODAS 2.0 was forward translated independently from English to Igbo by one clinical physiotherapist (native Igbo speaker, bilingual in Igbo and English) and one bilingual non-clinical translator (native Igbo speaker, bilingual in Igbo and English) to obtain two Igbo versions: T1 and T2 respectively. The forward translators were both fluent in English. The physiotherapist, a specialist in musculoskeletal physiotherapy, had all the items explained to her to facilitate an understanding of the construct being assessed to ensure psychometric equivalence with the original WHODAS 2.0. For the non-clinical translator, items were not defined to ensure that the language and expressions used in the translation reflected the routinely used language in the population.

Second step – a discussion between the two forward translators, mediated by the bilingual (English and Igbo) lead author resulted in the synthesis of T1 and T2 to produce one Igbo version: T-12. The two forward translated versions of the WHODAS were compared to the original questionnaire to inform their synthesis. The lead author compared the translations, noted, and recorded all discrepancies and discussions. The process of consensus between the translators was achieved through the analyses of the discrepancies and choosing the meaning that most closely reflected the original measure.

Third step – the synthesized Igbo version (T-12) was back translated from Igbo to English by two back non-clinical translators blinded to the original WHODAS 2.0 and the construct it measures, and were naïve in the disease involved. This produced two back-translated English versions: BT1 and BT2. One of the back translators was an English/Igbo linguistic expert proficient in the professional translation of tools, and the other was a native English speaker, born in England to Nigerian-born Igbo parents. This validation process ensured that the adapted measure was reflecting the meaning in the original WHODAS 2.0.

Fourth step – a pre-final Igbo version of the WHODAS 2.0 was produced following several meetings of the external expert review committee and translators during which all versions of the measure (T1, T2, T-12, BT1 and BT2) were discussed, mediated by the lead author.

The committee achieved semantic equivalence by exploring Igbo and English words of the same object to determine if they meant exactly the same thing; if the same terms could have several meanings; and if grammatical difficulties were encountered during the translations. The committee accomplished experiential equivalence with the original measure by ascertaining that items in both versions were experienced in the same way in the two cultures. The committee established that words in the instructions, items, and responses had comparable conceptual meanings in Igbo and English cultures [10]. The Igbo words used in the translations were simple enough to be understood by anyone regardless of their educational level.

Fifth step – twelve adults living with CLBP in a rural Nigerian community [9] pre-tested the pre-final Igbo-WHODAS 2.0. This number is sufficient for the qualitative assessment of the relevance, comprehensiveness and comprehensibility of the translated WHODAS 2.0 since the COSMIN checklist recommends a sample size of at least 7 participants [8]. The think-aloud cognitive interviewing procedure was used. This involved reading out each item. Participants then loudly verbalised their thoughts as they attempted to answer each question. Participants finally stated if they encountered any difficulty understanding any item, what they understood by each question, and the perceived meaning of their selected response(s). All responses were recorded verbatim. This procedure helped to maintain equivalence between the different settings ensuring face and content validity of the Igbo-WHODAS 2.0.

### Psychometric testing process

#### Participants (sample size calculation for test-retest reliability)

A minimum sample size of 27 is required to detect an intra-class correlation coefficient of 0.9 and a maximum width of 0.23 for a 95% confidence interval. A study for examining test-retest reliability was conducted with a convenience sample of 50 adults with CLBP who had no underlying serious pathology, radiculopathy or spinal stenosis. The participants were aged between 18 and 69 years. They were recruited from rural and urban communities in Enugu State, South-eastern Nigeria. Informed consent was duly obtained prior to participation in the study.

#### Participants (sample size calculation for construct validity)

A correlation coefficient of 0.2 at a level of 0.05 with a power of 80% would require a sample size of 194. In a dataset with several high factor loading scores (> 0.80), a sample size of 150 would be sufficient for exploratory factor analysis (EFA). A representative random sample of 200 adults with CLBP were recruited from rural communities in Enugu State as part of a larger population-based study [12]. Participants were screened to rule out underlying serious pathology, radiculopathy or spinal stenosis. Informed consent was obtained prior to participation in the study.

#### Procedure for psychometric testing

A significant proportion of rural dwellers in Nigeria are not literate. Therefore, community health workers (CHWs), the front line of rural Nigerian primary health care, were recruited and trained for interviewer-administration of the questionnaires. The training was daily, face-to-face, and group-based to minimise common survey errors. A representative sample of the population obtained through multistage cluster sampling prevented coverage error. An adequate sample size and gender stratification prevented sampling error. The use of validated measures and training CHWs to avoid administering the measures in ways that could bias participants’ responses reduced measurement error. Non-response error was avoided by ensuring that no items or scales were unanswered and that all recruited participants were assessed.

#### Collection and fidelity of data

CHWs screened participants by asking simple questions to exclude back pain due to malignancy, spinal fracture, infection, inflammation or cauda equina syndrome. They were then asked to describe the location of their pain with a body chart to confirm pain in the lower back. The WHODAS 2.0, Igbo-RMDQ and BS-11 were then interviewer-administered with Likert scales presented to participants as ‘flash cards’ as each corresponding item was read out. ‘lower back/waist pain’ was read out to participants in place of ‘illness’. The BPS was objectively used to assess performance-based disability.

For test-retest reliability, measures were completed at baseline and repeated 7 to 10 days post-baseline, with the same CHW collecting data on the two occasions.

To test validity, measures were completed at one time-point in a cross-sectional design.

Fidelity checks were done to avoid systematic differences in data collection. The CHWs were given post-training examinations, and only those that passed them were recruited. This facilitated adherence to data collection protocols. Additionally, each CHW was visited by the lead author during data collection without prior notice to assess their data collection and recording.

#### Data analyses

IBM Statistical Package for Social Sciences version 22 (SPSS, Chicago, IL) was utilised. Visual (normal distribution curve and Q-Q plot), and statistical (Kolmogorov-Smirnov, Shapiro-Wilk’s test and Skewness/Kurtosis scores) methods for assessing normality of data were employed.

##### Reliability

Reliability is the ability of an instrument to measure consistently. Test–retest reliability evaluated how consistently the adapted WHODAS 2.0 consistently measured disability over time using intra-class correlation coefficient (ICC). ICC was calculated using a two-way random effects model (measurement errors arising from either raters or subjects), using an absolute agreement definition between test-retest scores. 0.7, 0.8 and 0.9 signified good, very good and excellent ICCs [13]. Internal consistency (Cronbach’s alpha) depicts the extent to which all items in a test measure the same construct and was rated as weak (0–0.2), moderate (0.3 0.6) and strong (0.7–1.0) [14]. Bland-Altman plots, (which accounted for the weakness of ICC which might indicate strong correlations between two measurements with minimal agreement) were employed to visually assess the agreement level between test-retest measurements by plotting mean scores against difference in total scores. Standard error of measurement (SEM) and minimal detectable change (MDC) were also used to investigate reliability. MDC is a statistical estimate of the smallest change an instrument can detect which signifies a noticeable change which is not due to measurement error. MDC was calculated with the standard error of measurement (SEM), based on the distribution method, and the reliability of the measure [15]. SEM was based on the standard deviation (SD) of the sample and the test-retest reliability (R) of the Igbo-WHODAS 2.0, and was calculated with the equations [16]:

$$ \mathrm{SEM}=\mathrm{SD}\;\sqrt{\left(1-\mathrm{R}\right)} $$

MDC was then estimated with the equation:

$$ \mathrm{MDC}=1.96\ast \sqrt{2}\ast \mathrm{SEM} $$

1.96: 95% confidence interval of no change;

√2: two assessments used in determining change.

##### Validity

Construct validity assesses the extent to which a measure evaluates the construct it was intended to measure. The domain of construct validity assessed was convergent validity, which assesses whether two measures of the same/similar construct that are assumed to be theoretically related, are in fact related. This was investigated using Spearman’s correlation (non-parametric data) and was rated as weak (0–0.2), moderate (0.3–0.6), and strong (0.7–1.0). The WHODAS 2.0 assesses self-reported disability within the ICF multiple domains of cognition, mobility, self-care, getting along with people, life activities and participation – including work-related disability. Hence, Igbo-WHODAS 2.0 is expected to correlate at least moderately with the Igbo-RMDQ (measuring self-reported back pain-related disability), the BPS (objective measure of performance-based disability), and the Igbo-BS-11 (self-reported pain intensity measure and a predictor of self-reported disability) [12, 17].

### Outcome measures for construct validity testing

#### Igbo Roland Morris disability questionnaire (Igbo-RMDQ)

Igbo-RMDQ is a valid and reliable measure of LBP disability that is simple to administer, easily understood, and is best for population or primary care-based studies. Igbo-RMDQ is a twenty-four item back specific self-report measure with possible scores of 0 or 1 for each item. A score of 24 is the highest possible disability level and 0 means that there is no disability. It has good face/content validity, construct validity, internal consistency, test-retest reliability and responsiveness. It has Cronbach’s alpha of 0.91; test-retest reliability of 0.84; and a 2–3-point change from baseline means clinical significance [2].

#### Back performance scale (BPS)

BPS is a back-specific performance-based measure of mobility-related limitation that is scored by an evaluator. It involves five tests. Sock test involves simulating putting on a sock normally from the sitting position. Pick-up test involves picking up a piece of paper from the floor normally. For the roll-up test, the participant rolls up slowly from supine lying to long sitting with both arms relaxed. Finger-tip-to-floor test involves standing on the floor with both feet 10 cm apart. There is then forward bending with straight knees. The person then attempts to touch the floor with the fingertips. The distance between the floor and the fingertips is then measured in centimetres. The lift test involves a participant repeating the lifting of a 5-kg box from the floor to a 76 cm table and back to the floor for 1 min. The number of lifts is then recorded. Each of the five tests has scores ranging from 0 to 3 depending on the difficulty or ease with which they are performed. A total possible score of 15 signifies maximum disability while 0 means no disability [18]. The BPS has internal consistency of 0.73; moderate correlations with self-reported back pain specific disability (r = 0.454), and test-retest reliability of 0.91 [18, 19].

#### Eleven-point box scale (BS-11)

BS-11 is a single item eleven-point numeric scale for pain intensity [20]. It consists of eleven numbers (0 to 10) in boxes. Zero means ‘no pain’ and 10 is ‘pain as bad as you can imagine’ or ‘worst pain imaginable’. The measure is more easily understood than the visual analogue scale in this population [9].

Exploratory factor analyses (EFA) was used to determine the number of factors influencing the Igbo-WHODAS (the items that go together – dimensionality). EFA was applied in line with the Kaiser Meyer Olkin (KMO) and the Bartlett’s test with eigenvalue for retention set at ⩾1.0 (Kaiser’s rule) [21]. Retained and excluded factors were also explored visually on a Scree plot. Promax (oblique) rotation, which assumes that factors can be related, was done, and factor loadings less than 0.3 were suppressed. Extraction was done using principal axis factoring. The number of factors and the fundamental relationships between the items were then compared with the factor structures of the original WHODAS 2.0 to augment any insight of possible differences in population characteristics.

#### Floor and ceiling effects

When a high proportion of participants score the highest or the lowest score, ceiling or floor effect respectively occurs. This implies that a measure is unable to discriminate between either extreme of the scale. A ceiling or floor effect was defined as 15% or more of the total sample of 250 participants scoring 0 or 100 on the Igbo-WHODAS 2.0 [22].

## Results

### Participant characteristics

Table 2 highlights the socio-demographic characteristics of all the participants that participated in this study (cross-cultural adaptation, test-retest reliability and construct validity samples).

**Table 2:** Demographic characteristics of all participants (cross-cultural adaptation, test-retest reliability and construct validity samples)

	AGE	GENDER	MARITAL STATUS	MAIN OCCUPATION	RELIGION	EDUCATION (years completed)	LITERACY	HABITATION
Cross-cultural adaptation (pilot/pre-testing) sample; *n* = 12	45 years (SD 10.36)	Male: 7 (58.3%)	Married: 11 (91.7%) Single: 1 (8.3%)	Non-manual workers: 5 (41.7%) Manual workers: 7 (58.3%)	Pentecostal: 10 (83.3%) Catholic: 2 (16.7%)	10.0 (3.7)	Illiterate: 4 (33.3%) English: 6 (50%) English/Igbo: 2 (16.7%)	Rural
Test-retest reliability sample; *n* = 50	45.2 years (SD 11.55)	Male: 18 (36.0%)	Married: 37 (74.0%) Single: 8 (16.0%) Widowed: 4 (8.0%) Separated: 1 (2.0%)	Paid Non-manual: 25 (50.0%) Self-employed business/farming: 19 (38.0%) Keeping house/homemaker: 2 (4.0%) Student: 2 (4.0%) Non-paid work/volunteer/charity: 1 (2.0%)		13.3 (7.14)		Urban: 30 (60.0%) Rural: 20 (40.0%)
Construct validity sample; *n* = 200	48.6 years (SD 12.0)	Male: 112 (44.0%)	Married: 143 (71.5%) Widowed: 31 (15.5%) Single: 22 (11.0%) Cohabiting: 2 (1.0%) Separated: 2 (1.0%)	Self-employed business/farming: 125 (62.5%) Paid Non-manual: 31 (15.5%) Non-paid work/volunteer/charity: 16 (8.0%) Keeping house/homemaker: 13 (6.5%) Student: 7 (3.5%) Unemployed (health reasons): 4 (2.0%) Unemployed (other reasons): 3 (1.5%) Retired: 1 (0.5%)		7.0 (6.4)		Rural: 200 (100%)

### Translation, comprehensibility, comprehensiveness and cultural equivalence of Igbo-WHODAS

The expert committee retained interviewer instructions in English in the Igbo-WHODAS 2.0 (Supplemental file 1) as the interviewers were literate, and evidence from this population suggests that literate people found it easier to read English than Igbo [9]. Patient instructions were meant to be read out to participants, and these were translated and cross-culturally adapted into Igbo. The committee added ‘waist pain/lower back pain’ to ‘illness(es)’ to make the measure back pain-specific for this study whilst allowing the measure to be used for other conditions in future studies. In item D1.3, the forward translators wrote ‘understanding and finding out solutions’ as a translation of ‘analysing and finding solutions’. This was modified to the Igbo equivalent of ‘probing/exploring/researching’ and ‘finding out/discovering solutions’ by the expert review team to better reflect the original item as there is no Igbo word for ‘analyse’. The Igbo phrase for ‘people close to you’ also means ‘people near you’. The latter would not reflect the original item D4.3. Therefore, translators used the Igbo phrase for ‘family and friends’ to better represent ‘people close to you’. For item D6.1, forward translators translated ‘how you do things in your community’. Discrepancy was detected after back translation; hence the phrase was changed to ‘…in joining in activities that are performed in your community…’ by the translation and expert review committee to better reflect the original item. ‘…affected your heart or spirit’ was used in place of ‘emotionally affected’ in item D6.5 as there is no Igbo word for emotion. ‘Deplete or affect’ was used in place of ‘drain’ in item D6.6. ‘To what extent’ was used in place of ‘how much’ throughout the measure to better reflect the original items through consensus of the translators and the expert review committee. This is because ‘how much’ could also be understood as ‘how many’ in Igbo. All modifications are in Supplemental file 2.

### Psychometric properties of Igbo-WHODAS

#### Findings from fidelity assessment

The CHWs strictly adhered to the interviewing styles recommended during their training. They remained neutral throughout the interviews. They did not react verbally or nonverbally to participants’ responses. They discouraged participants’ digression, distraction and inappropriate enquiries. They maintained the wording and sequence of questions in the measures and recorded data as appropriate. They provided only one answer to each item, written in the space provided for each item in each measure. Their assessment of performance-based disability was adequate, as they used tape measures adequately to assess 10 cm between the feet and measured the distance between the fingertips and the floor, for the finger-tip-to-floor test. The performance-based disability levels recorded by the first author and the CHWs were found to be similar for the few participants randomly selected.

#### Reliability

Internal consistency, intraclass correlation coefficients, standard error of measurements and minimal detectable changes, for the total score and each subscale are presented in Table 3. Cronbach’s alpha if each of the items is deleted in the total score and in each of the subscales is presented in Supplemental file 3. Acceptable agreements were found between test-retest values of the Igbo-WHODAS and its subscales as mean differences were close to zero and most points were within the 95% limits of agreement of the mean differences (Supplemental file 4).

**Table 3:** Reliability of Igbo-WHODAS

**Igbo-WHODAS total score** Number of items: 36; Cronbach’s alpha global score: 0.97; ICC (95% CI): 0.93 (0.88, 0.96)	
SEM: 5.05 MDC: 13.99	
**Igbo-WHODAS 2.0 (cognition)** Number of items: 6; Cronbach’s alpha global score: 0.88; ICC (95% CI): 0.87 (0.77, 0.93)	
SEM: 7.20 MDC: 19.96	
**Igbo-WHODAS 2.0 (mobility)** Number of items: 5; Cronbach’s alpha global score: 0.91; ICC (95% CI): 0.90 (0.83, 0.94)	
SEM: 8.00 MDC: 22.17	
**Igbo-WHODAS 2.0 (self-care)** Number of items: 4; Cronbach’s alpha global score: 0.75; ICC (95% CI): 0.82 (0.68, 0.90)	
SEM: 7.20 MDC: 20.35	
**Igbo-WHODAS 2.0 (getting along with people)** Number of items: 5; Cronbach’s alpha global score: 0.81; ICC (95% CI): 0.81 (0.66, 0.89)	
SEM: 7.20 MDC: 20.35	
**Igbo-WHODAS 2.0 (life activities)** Number of items: 8; Cronbach’s alpha global score: 0.95; ICC (95% CI): 0.93 (0.87, 0.96)	
SEM: 8.70 MDC: 24.11	
**Igbo-WHODAS 2.0 (participation)** Number of items: 8; Cronbach’s alpha global score: 0.92; ICC (95% CI): 0.85 (0.73, 0.91)	
SEM: 11.10 MDC: 30.77	

*ICC* Intraclass correlation coefficient, *SEM* Standard error of measurement, *MDC* Minimal detectable change, *CI* Confidence interval

### Construct validity

Table 4 illustrates the total scoring of the Igbo-WHODAS and its subscales which correlated moderately (rs ≥ 0.3) with performance-based disability (BPS), self-reported disability (Igbo-RMDQ), and pain intensity (BS-11), except for the cognition and getting along subscales. There was a weak (rs =0.19) but statistically significant correlation between the cognition subscale of the Igbo-WHODAS and performance-based disability. There was no correlation between the getting along subscale of the Igbo-WHODAS and performance-based disability.

**Table 4:** Spearman’s correlation between Igbo-WHODAS and self-reported back pain-specific disability, performance-based disability and self-reported pain intensity

	Igbo-RMDQ	BPS	BS-11
Igbo-WHODAS total	0.54**	0.34^**^	0.56**
Igbo-WHODAS cognition	0.31**	0.19^**^	0.44**
Igbo-WHODAS mobility	0.60**	0.35^**^	0.50**
Igbo-WHODAS self-care	0.39**	0.28^**^	0.25**
Igbo-WHODAS getting along	0.29**	0.09	0.31**
Igbo-WHODAS life activities	0.46**	0.33^**^	0.54**
Igbo-WHODAS participation	0.50**	0.36^**^	0.55**

**p < 0.01

**Fig. 1:**
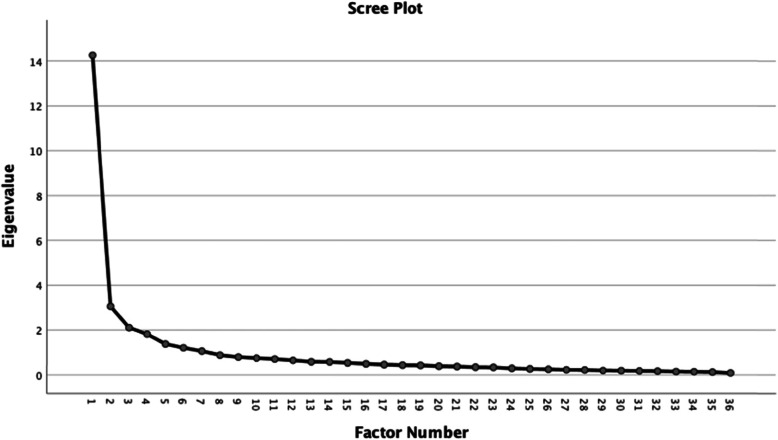
Scree plot of Igbo-WHODAS 2.0 (total score)

A scree plot in Fig. 1 suggests a seven-factor structure of the Igbo-WHODAS; which is corroborated in Table 5 with 62.79% of the items having factor loadings above 0.5; and 66.67% of the items loading on the corresponding factor in the original measure. Factor 1 contains all the items of the original life (household and work/school) activities subscale in addition to two items of the original participation subscale (problem joining in community activities (D6.1), and problem doing things by oneself for relaxation/pleasure (D6.8); and one item of the original self-care subscale (staying by oneself for a few days).

**Table 5:** Exploratory factor analysis of the Igbo-WHODAS

***n*** = 200	1	2	3	4	5	6	7
WHODAS D5.4	.904						
WHODAS D5.1	.846						
WHODAS D5.2	.799						
WHODAS D5.8	.790						
WHODAS D5.7	.730						
WHODAS D5.3	.724						
WHODAS D5.5	.720						
WHODAS D5.6	.704						
WHODAS D6.1	.503						
WHODAS D3.4	.421						
WHODAS D6.8	.305						
WHODAS D4.4		.897					
WHODAS D4.1		.812					
WHODAS D4.3		.680					
WHODAS D4.2		.549					.364
WHODAS D6.3		.503					.332
WHODAS D1.5		.484		.377			
WHODAS D4.5		.413					
WHODAS D2.4			.809				
WHODAS D2.2			.720				
WHODAS D2.5			.640				
WHODAS D2.1			.624				
WHODAS D2.3			.584				
WHODAS D6.4			.546				
WHODAS D6.5			.477				
WHODAS D1.2				.759			
WHODAS D1.1				.713			
WHODAS D1.3				.680			
WHODAS D1.4				.473			
WHODAS D1.6		.411		.459			
WHODAS D6.6					.912		
WHODAS D6.7					.828		
WHODAS D3.1			.310			.737	
WHODAS D3.2						.689	
WHODAS D3.3		.456				.480	
WHODAS D6.2				.408			.421
KMO = 0.92 χ^2^ = 4984.50***							

Only factor loadings above 0.3 are shown; KMO = Kaiser-Meyer-Olkin measure of sampling adequacy; χ^2^ = Bartlett’s test of sphericity tested with chi-square ***p < 0.001; Extraction Method: Principal Axis Factoring; Rotation Method: Promax with Kaiser Normalization; Rotation converged in 11 iterations.

Factor 2 contains all the items in the original getting along subscale in addition to one item of the original participation subscale D6.3 (living with dignity), and one item of the original cognition subscale D1.5 (understanding what people say).

Factor 3 matches the mobility subscale of the original measure, but with two additional items from the original participation subscale (time spent on back pain and emotional effects of back pain) loading on it. Factor 4 corresponds to the cognition subscale of the original measure except that one of the items in the original subscale (understanding what people say) loaded on the getting along factor. Factor 5 (participation subscale) had only two items of the original subscale, loading on it (back pain drained financial resources and back pain caused family problems). It was the least precise subscale as items from the original participation subscale loaded on all factors except the self-care factor. Factor 6 matches the self-care subscale of the original measure except for one missing item (staying by yourself for a few days) that loaded on the life activities factor. Factor 7 had only one major item (barriers and hindrances in the world around one due to back pain) from the original participation subscale (Table 5).

### Floor and ceiling effects

Eight (3.2%) participants scored 0 and no one (0%) scored 100 on the Igbo-WHODAS. 72 (28.8%) participants scored 0 on the cognition subscale, 27 (10.8%) scored 0 on the mobility subscale, 86 (34.4%) scored 0 on the self-care subscale, 62 (24.8%) scored 0 on the getting along subscale, 17 (6.8%) scored 0 on the life activities subscale, 21 (8.4%) scored 0 on the participation subscale; but no one (0%) scored 100 on any of the subscales. The Igbo-WHODAS 2.0 and its subscales did not have ceiling effect. However, floor effect was observed in cognition, self-care and getting along subscales.

## Discussion

### Summary of main findings

This study enabled the cross-cultural adaptation and psychometric evaluation of the 36-item interviewer-administered version of the WHODAS 2.0 for Igbo speaking populations. The WHODAS 2.0 was straight forward to cross-culturally adapt, comprehend and was acceptable. The cross-cultural adaptation confirmed its face and content validity.

Igbo-WHODAS and its subscales demonstrated adequate reliability, agreement and construct validity. It had good internal consistency (α = 0.75–0.97); intra class correlation coefficients (ICC = 0.81–0.93); standard error of measurements (5.05–11.10) and minimal detectable change (13.99–30.77). Acceptable agreement levels were found between the test-retest values of the Igbo-WHODAS and its subscales. The measure and its subscales correlated at least moderately (rs ≥ 0.3) with performance-based disability, self-reported back pain specific disability (Igbo-RMDQ), and pain intensity (BS-11), except for the cognition and getting along subscales. There was a weak (rs =0.19) but statistically significant correlation between the cognition subscale of the Igbo-WHODAS and performance-based disability. There was no correlation between the getting along subscale of the Igbo-WHODAS and performance-based disability.

A seven-factor solution of the Igbo-WHODAS was produced in contrast to the six factors in the original measure [5]. Most Igbo-WHODAS items loaded on their corresponding factor in the original measure except for participation. The participation subscale of the original WHODAS 2.0 (meant to reflect the impact of participants’ back pain on their participation in society) was the least precise with only two of the original eight items (‘drain on financial resources’ and ‘problem to family’) loading on factor 5. The other items in the original participation subscale loaded on all other factors except self-care. Differences could be due to high illiteracy resulting in high measurement error or different population characteristics. The latter is more likely to be the case.

The Igbo-WHODAS 2.0 did not have floor and ceiling effects although floor effects were observed in the cognition, self-care and getting along subscales.

### Strengths and limitations addressing potential sources of bias and imprecision

The strengths of this study include good comprehensibility and acceptability of the Igbo-WHODAS; validation of Igbo-WHODAS 2.0 with both self-reported and performance-based disability as well as pain intensity measures, with correlations which are in line with the established literature, supporting its validity.

The exact Igbo equivalents of some English words were lacking which was resolved by using Igbo phrases that retained the conceptual meaning in the original items. This could be because Igbo language may be more adapted to colloquial speech than scientific writing [23]. English is the official written language in Nigeria which may explain why literate Igbo Nigerians prefer to read/write English but speak Igbo informally. It was found that some Igbo words/phrases had multiple meanings depending on the context, which was resolved by using Igbo phrases with all possible meanings reflecting the original items.

### Interpretation of the results in comparison with the current literature

The straight forward cross-culturally adaptation, easy comprehensibility and good acceptability of the Igbo-WHODAS concurs with previous adaptations [15, 24, 25]. Cronbach’s alpha of Igbo-WHODAS and its subscales ranging between 0.75–0.97 concurs with the original measure [5], and other adaptations [25–27]. However, the Cronbach’s alpha was slightly higher in the original measure possibly due to different population characteristics such as literacy. Igbo-WHODAS and its subscales demonstrated reliability with ICCs that were very good to excellent (0.81–0.93). The good agreement shown in the Bland-Altman plots mirrors the original measure [5], and other adaptations [25, 27]. The seven-factor solution of the Igbo-WHODAS is similar to its European [27] and Chinese [25] versions.

The lack of association between the getting along subscale of the Igbo-WHODAS and performance-based disability could be because the getting along with people subscale appears to reflect the psychosocial aspect of the biopsychosocial disability model whereas the back-performance scale measures the biomedical aspect of the biopsychosocial disability model. In contrast to the Igbo-WHODAS which fully captures the multidimensional biopsychosocial disability concept including impairments, activity limitations and participation restrictions; performance-based disability is impairment focused. Impairment represents abnormalities or loss of body structure and function and conceptualises disability at the level of the body only [28]. Impairment does not automatically imply disability, as people with impairment may not experience disability, or do so at varying levels depending on personal, physical and social barriers/facilitators in different contexts [29]. Evidence suggests that performance-based disability characterise impairment-focused biomedical variables (e.g. leg strength, leg velocity), whereas patient-reported disability represents both impairment and psychosocial aspects of disability [30]. This agrees with our findings showing the greatest correlations between Igbo-WHODAS, its mobility, participation, and life activities subscales; and back pain specific disability (Igbo-RMDQ) and pain intensity (BS-11) which are patient-reported outcomes.

Furthermore, these subscales represent the construct represented within established back pain specific measures. Cognitive dysfunction may be less important than limitations in mobility, life activities (difficulties in performing specific actions, tasks or activities related to household activities and work/school activities) and participation (difficulties in participating in community activities within the society) in this population. As expected, the mobility subscale of the Igbo-WHODAS had one of the strongest correlations with the BPS which measures mobility-related disability [18]. These findings support the construct validity of the Igbo-WHODAS 2.0.

The floor effects observed in the cognition, self-care and getting along subscales of the Igbo-WHODAS could mean that these are not the major domains affected in CLBP-disability in rural Nigeria. Pain intensity, mobility, work activities and participation may be the most affected [9, 12].

### Consideration of clinical and scientific implications of the findings

The lack of an Igbo word for ‘emotion’ in item D6.5 may reflect the unclear emotional concept in this culture where emotional distress is often expressed through somatisation [9, 31]. This has been found in other non-western settings [32, 33]. ‘Affected your heart or spirit’ was therefore used to achieve conceptual equivalence.

Regarding the appropriateness of the SEM and MDC, 19% (Japan) to 51% (Nigeria) reduction in WHODAS is clinically important [5]. This corresponds to between 4.8 and 12.97 of Igbo-WHODAS mean of 25.44. Therefore, SEM of 5.05, MDC of 13.99 and limits of agreement of − 8.58 to 9.54 of Igbo-WHODAS appear suitable.

Factor 1 of the Igbo-WHODAS can be termed life activities, community involvement and functional independence factor as it reflects the difficulties participants may have in: performing daily household/work/school activities, joining in community activities, doing things or staying by oneself. The rural dwellers from whom the factor structure of the Igbo-WHODAS was derived were mostly involved in informal self-employed occupations within the community [2, 9, 12]. This could explain why work activities, community involvement and staying/doing things for oneself loaded as one factor. Factor 2 of the Igbo-WHODAS can be retained as the getting along factor as in the original subscale. The additional loading of one item of the original participation subscale (D6.3) and one item of the original cognition subscale (D1.5) suggests that living with dignity due to the action of others and understanding what people say are key to participants getting along with others in the community.

Factor 3 of the Igbo-WHODAS can be named mobility and concern factor since two additional items from the original participation subscale (time spent on back pain D6.4, and emotional effects of back pain D6.5) loaded on it. This suggests that participants are less likely to be mobile when they spend time worrying about their back pain. This concurs with qualitative results from this population showing that people with CLBP often spend time alone in bed thinking and worrying about their condition [9]. This may explain why the two items ‘time spent on back pain’ and emotional effects of back pain loaded together. The designation for factor 4 can be retained as cognition as in the original cognition subscale despite one of the original items (understanding what people say D1.5) loaded on the getting along factor. Understanding what people say may be more important to getting along with people than cognition in this population.

Factor 5 can be termed financial impact as it had only two items (back pain drained financial resources D6.6 and back pain caused family problems D6.7) in the original participation subscale loading on it. Qualitative research evidence [9] from this population suggests that reduction of financial resources due to work-related disability from CLBP had adverse effects on family relationships as indicated by participant comments:

“…It means that you are not able to do the work that supports your existence. With that you will see that there will be no money, there will be no food until I recover and start going to work…” (P3, Male, aged 42 years).

“…brings problems into the home…because the money isn’t enough…“(P17, Male, aged 46 years) [9].

Factor 6 is entitled self-care as in the original self-care subscale despite having one missing item (staying by yourself for a few days D3.4) that loaded on factor 1 (life activities, community involvement and functional independence factor). Notably, this item D3.4 in the original self-care subscale appears very similar to item D6.8 problem doing things by oneself for relaxation/pleasure in the original participation subscale. These concepts appear to belong to one construct and should be examined in future studies. Factor 7 can be regarded as redundant as it had only one major item D6.2 (barriers and hindrances in the world around an individual due to back pain) from the original participation subscale. However, factor 7 had secondary loadings from two items, D6.3 (problem living with dignity due to attitudes/actions of others) and D4.2 (difficulties maintaining a friendship), both of which loaded primarily on factor 2 (getting along with people). This suggests that the barriers and hindrances that people with CLBP face in rural Nigeria could be related to problems they have living with dignity due to attitudes/actions of others and difficulties maintaining a friendship. These findings require further exploration. Moreover, further research is required to confirm the factor structure of the Igbo-WHODAS.

### Future research and unanswered questions

Despite acceptable validity and reliability levels, high sample variability and measurement errors were present, possibly introduced by low literacy rates, interviewer-administration and data collection by several raters. This is important as MDC not only depends on the inherent measurement error of an instrument, but varies across populations and contexts [34, 35]. Hence, sensitivity-to-change studies of the Igbo-WHODAS 2.0 is required in populations of varying literacy levels, with single raters, and using more rigorous analysis such as receiver operating characteristic (ROC) curves, which includes patients’ own global impression of change [36]. Furthermore, these studies need to confirm the MDC of the Igbo-WHODAS and determine the proportion of people that achieve it.

Bilingual assessment of the agreement between the original WHODAS and Igbo-WHODAS 2.0, including item by item agreement was not performed. This is necessary in future studies of the Igbo-WHODAS 2.0 and should involve a population with adequate literacy levels to enable comprehension of the English and Igbo versions.

The lack of rigorous investigation of item redundancy in this study can be explored in future studies. Redundancy could be demonstrated in terms of items that are too similar which spuriously inflate reliability [37], or items that are not applicable in this particular culture or population [38]. Reducing redundancy involves excluding items that are not applicable in a population following assessment by a team of content experts from a culture. Items rated by a single team member as irrelevant, or by two or more members as questionably relevant is usually eliminated. In contrast, items obtaining one rating of questionable relevance are reconsidered for inclusion. Re-assessment of internal consistency is then needed when any item is removed from a measure to ensure that an acceptable Cronbach’s alpha (> 0.60) is maintained [39].

Following the elimination of redundancy, multi-group confirmatory factor analyses may be needed to compare and determine the factor structures of the Igbo-WHODAS with the best fit indices in rural Nigeria; assess if the same items assess the same construct in different populations in rural Nigeria; investigate whether the items of a given factor are equally significant within different cultures in rural Nigeria or are too different; and if items are more biased towards some cultural groups than others. Using the item response theory, the items of the Igbo-WHODAS with different functioning may be eliminated so that groups are comparable, in which case the Igbo-WHODAS may become slightly different from the original WHODAS or the current version of the Igbo-WHODAS may be considered differently in separate groups to maintain equivalence between scores [37].

The acceptable internal consistency of the Igbo-WHODAS 2.0 suggest that items were sufficiently independent but were adequately similar. However, Principal Components Analysis (PCA), a data reduction technique which identifies and discards highly correlated items may be required in future studies of the Igbo-WHODAS 2.0. As PCA is a large sample evaluation requiring at least five times the number of items in a questionnaire being analysed, a much larger sample size than the one used in this study would be required. This is more so when only a few items are expected to load onto each component, and when variable communalities (percentage of variance in an observed variable that is accounted for by the retained components) are low [39]. Furthermore, confirmatory factor analyses of the Igbo-WHODAS 2.0 is required in future studies. This would require a sample size of at least 300 when there are only a few high factor loading scores (> 0.80) [40].

## Conclusions

The Igbo-WHODAS appears valid and reliable. Further rigorous testing is required to establish its utility for clinical and research purposes in Igbo speaking culture.

## Supplementary Information

**Additional file 1.** The Igbo World Health Organisation Disability Assessment Schedule (Igbo-WHODAS 2.0).

**Additional file 2.** Changes made to Igbo-WHODAS following cross-cultural adaptation.

**Additional file 3:.** Reliability of Igbo-WHODAS.

**Additional file 4.** Bland-Altman plots of the Igbo-WHODAS.

## Data Availability

Data is available on request due to ethical restrictions imposed by Biomedical & Health Sciences, Dentistry, Medicine and Natural & Mathematical Sciences Research Ethics Subcommittees (BDM RESC) Kings College London. Requests for data access may be made to BDM RESC Kings College London through email bdm@kcl.ac.uk.
