# Supplementary material for: World Health Organisation Disability Assessment Schedule (WHODAS 2.0): development and validation of the Nigerian Igbo version in patients with chronic low back pain
Source: BMC Musculoskelet Disord. 2020 Nov 17;21:755. doi: 10.1186/s12891-020-03763-8 (PMC7670680; doi:10.1186/s12891-020-03763-8)
Supplement: Supplementary file 1 — Additional file 1. The Igbo World Health Organisation Disability Assessment Schedule (Igbo-WHODAS 2.0). [file 12891_2020_3763_MOESM1_ESM.pdf]

IGBO-WHODAS 2.0 36-ITEM VERSION, INTERVIEWER-ADMINISTERED (Igbo World Health Organisation Disability Assessment Schedule)

Instructions to the interviewer are written in bold and italics-do not read these aloud.

Text for the respondent to hear is written in standard print in blue and in Igbo.

Read this text aloud

Section 1: Face sheet

Complete items F1-F5 before starting each interview				
F1	Respondent identity number			
F2	Interviewer identity number			
F3	Assessment time point (1, 2, etc.)			
F4	Interview date	Day.................	Month.......	Year......
F5	Living situation at time of interview (circle only one)	Independent in community	1	
		Assisted living	2	
		Hospitalised	3	

Section 2: Demographic and background information

Ajuju onu a bu ndi World Health Organisation (WHO) weputara ya ka ewere ghota nsogbu ndi mmadu na enwe gbasara onodu ahu ike ha (ukwu mgbu). Aziza gi niile no na akwukwo a bu naani maka iji wee mee ihe nyocha a burukwa ihe agaghi egosi ndi ozo. Ajuju a ga ewe nkeji iri na ise ma o bu iri abuo iji wee zachaa ya.

For respondents from the general population (not the clinical population) say:

O burugodi na inweghi ahuike ma o bu na inweghi nsogbu obula, e kwesiri m ijucha ajuju nile a, iji wee mee ka ihe nyochaa zuo oke.

Aga m amalite iju ajuju ndabere iji malite.

A1	***Record sex as observed***	Female<br>Male	1<br>2
A2	Afo ole ka i di ugbua?	...............years	
A3	Afo ole ka owere gi igu akwukwo na ulo akwukwo o bula, koleji ma o bu mahadum?	................years	
A4	Gini bu onodu alumdi na nwanyi gi ugbua?<br>***(select the single best option)***	Anughi m di ma o bu nwunye	1
		Ano m na anu m di na nwunye	2
		A nuru m di ma o bu nwunye mana mu na ya ebighizi	3
		Mu na di ma o bu nwunye m gbara alukwaghi m	4
		Di m ma o bu nwunye m nwuru anwu	5
		Mu na enyi m nwoke ma o bu nwanyi bi	6
A5	Kedu uzo ka mma aga eji kowaa oru gi?<br>***(select the single best option)***	Oru a na akwu m ugwo	1
		E nwere m oru nke onwe m dika izu ahia ma o bu oru ugbo	2
		A na m aru oru anaghi akwu m ugwo	3
		Nwata akwukwo	4
		Ana m eledo ezi n'ulo m anya. Onweghi ihe ozo m na aru.	5
		A lara m ezumike nka	6
		Enweghi m oru (maka ahu ike m)	7
		Enweghi m oru (maka ihe ndi ozo)	8
		Ihe ndi ozo (biko kowaa)......................	9

Section 3: Preamble

Say to respondent:

Ajuju onu a bu maka nsogbu ndi mmadu na enwe na onodu ahu ike ha (dika ukwu mgbu).

Hand flashcard to #1 to respondent and say:

Gbasara onodu ahu ike a, ihe m na akowa bu nsogbu nile i na enwe gbasara oria gi (ukwu mgbu gi).

Chetakwa ka i buru onodu gbasara oria gi (ukwu mgbu gi) na obi ka i na aza ajuju ndi a. Mgbe m juo gi ajuju gbasara nsogbu i na enwe mgbe o bula i choro ime ihe, burukwa ihe ndi a na obi...

Point to flashcard #1 and explain that 'ihe ira ahu' putara:

- Igbawanye oke mbo
- Mmekpa ahu ma o bu ihe mgbu
- Ime ihe nwanyo
- Mgbanwe ma o bu ihe di iche na uzo isi eme ihe

Say to respondent:

Mgbe i na aza ajuju a, aga m acho ka iche echiche maka ubochi iri ato gara aga. Aga m acho ka izaa ajuju ndia nile ka i na echeta udi nsogbu nile inwegoro mgbe i na eme ihe ndi i na emebu etu isi emebu ha na mbu ka mgbe ubochi iri ato gara aga.

Hand flashcard #2 to respondent and say:

Jiri usoro onu ogugu ndia mgbe i na aza ajuju ndi a.

Read the scale aloud:

Onweghi/Odighi, obere, ogafeghi oke, o siri ike, ogafere oke/enweghi m ike ime.

Ensure that the respondent can easily see the flashcards #1 and #2 throughout the interview

Section 4: Domain reviews

Domain 1: Cognition

Ugbu a, aga m aju gi ajuju gbasara etu isi aghota ihe na etu isi agwa mmadu okwu.

Show flashcards #1 and #2 to respondent

Na ubochi iri ato gara aga, kedu ihe isi ike i na enwe na ihe ndia:		Onweghi/Odighi	obere	ogafeghi oke	o siri ike	ogafere oke/enweghi m ike ime
D1.1	Echiche gi idigide na ihe i na eme ruo nkeji iri?	1	2	3	4	5
D1.2	Icheta ime ihe ndi di mkpa?	1	2	3	4	5
D1.3	i nyocha na ichoputa usoro iga eji gbo mkpa diri gi ubochi kwa ubochi?	1	2	3	4	5
D1.4	imuta ihe ohuru, dika imuta etu I ga esi aga ebe I gabeghi mbu?	1	2	3	4	5
D1.5	I ghota ihe nile ndi mmadu na ekwu?	1	2	3	4	5
D1.6	I malite na inogide na mkparita uka?	1	2	3	4	5

Domain 2: Mobility

Ajuju m ga aju gi ugbu a bu maka nsogbu I na enwe na ikpaghari.

Show flashcards #1 and #2

Na ubochi iri ato gara aga, kedu ihe isi ike I na enwe na ihe ndia:		Onweghi/Odighi	obere	ogafeghi oke	o siri ike	ogafere oke/enweghi m ike ime
D2.1	Ikwuru oto tee aka dika nkeji iri ato?	1	2	3	4	5
D2.2	Ikwuru oto mgbe inoduchara ala?	1	2	3	4	5
D2.3	Igaghari agaghari na ime ulo gi?	1	2	3	4	5
D2.4	Isi na ulo gi puo apuo?	1	2	3	4	5
D2.5	I ga ije tere aka dika otu kilometa (ma o bu ihe dika ya)?	1	2	3	4	5

Domain 3: Self-care

Ajuju m ga aju gi ugbu a bu maka ihe isike I na enwe maka ilekota onwe gi anya.

Show flashcards #1 and #2

Kemgbe ubochi iri ato gara aga, kedu nsogbu I na enwe ime ihe ndi a:		Onweghi/Odighi	obere	ogafeghi oke	o siri ike	ogafere oke/enweghi m ike ime
D3.1	Isa ahu gi nile?	1	2	3	4	5
D3.2	Iyinye akwa?	1	2	3	4	5
D3.3	Iri nri?	1	2	3	4	5
D3.4	Ino nani gi ubochi ole na ole?	1	2	3	4	5

Domain 4: Getting along with people

Ajuju m ga aju gi ugbua bu maka ihe isike I na enwe gi na ndi mmadu imekorita. Biko cheta na o bu soso ihe isike maka ihi oria gi (ukwu mgbu gi). Ihe m na akowa bu nsogbu gbasara oria gi (ukwu mgbu gi).

Show flashcards #1 and #2

Kemgbe ubochi iri ato gara aga, kedu nsogbu I na enwe ime ihe ndi a:		Onwegh i/Odighi	obere	ogafeghi oke	o siri ike	ogafere oke/enweghi m ike ime
D4.1	Imeso ndi I maghi omume?	1	2	3	4	5
D4.2	Inogide na enyi gi na ndi ozo nwere?	1	2	3	4	5
D4.3	Ihe I ga nke oma na etiti ndi gi na ha di na mma, ya na ezi n'ulo gi?	1	2	3	4	5
D4.4	Imete enyi ohuru?	1	2	3	4	5
D4.5	Mmekorita nwoke na nwanyi?	1	2	3	4	5

Domain 5: Life activities

5(1): Household activities

Ugbu a, aga m aju gi ihe gbasara ihe ndi I na eme iji akwado ezi n'ulo gi, na ilekota ndi gi na ha bi ma o bu ndi no gi na akuku. Ihe ndi a gunyere isi nri, ihicha ulo, izuta ihe na ahia, ilekota mmadu na ilekota ihe ndi i nwere.

Show flashcards #1 and #2

N'ihi oria gi (ukwu mgbu gi), kamgbe ubochi iri ato gara aga, kedu nsogbu i na enwe ime ihe ndi a:		Onweghi/ Odighi	obere	ogafeghi oke	o siri ike	ogafere oke/en weghi m ike ime
D5.1	Ilekota oru diri gi na ezi n'ulo gi?	1	2	3	4	5
D5.2	Ime ihe diri gi, ndi kachasi mkpa na ezi na ulo gi nke oma?	1	2	3	4	5
D5.3	Irucha oru nile I kwesiri iru na ezi n'ulo gi?	1	2	3	4	5
D5.4	I gbali ihu na aruchara oru diri gi na ezi na ulo gi ososo ka okwesiri?	1	2	3	4	5

If any of the responses to D5.2-D5.5 are rated greater than none (coded as "1"), ask:

D5.01	Kamgbe ubochi iri ato gara aga, ubochi ole ka ibelatara oru ezi n'ulo ma o bu hapu kpam kpam oru ezi n'ulo gi n'ihi oria gi (ukwu mgbu gi)?	***Record number of days........***

If the respondent works (paid, non-paid, self-employed) or goes to school, complete questions D5.5-D5.10 on the next page. Otherwise, skip to D6.1 on the following page.

5(2): Work or school activities

Ugbu a, aga m aju gi ajuju gbasara oru gi ma o bu ihe ndi i na eme na ulo akwukwo

Show flashcards #1 and #2

N'ihi oria gi (ukwu mgbu gi), na ubochi iri ato gara aga, kedu ka osi raa gi ahu ime ihe ndi a:		Onweghi/Odighi	obere	ogafeghi oke	o siri ike	ogafere oke/enweghi m ike ime
D5.5	Oru diri gi ubochi kwa ubochi na ulo oru/ulo akwukwo gi?	1	2	3	4	5
D5.6	Ime ihe ndi diri gi kachasi mkpa na ulo oru gi/ulo akwukwo gi nke oma?	1	2	3	4	5
D5.7	Irucha oru nile ikwesiri iru?	1	2	3	4	5
D5.8	Irucha oru gi ngwangwa ka okwesiri?	1	2	3	4	5
D5.9	I wetunatara aka na oru gi maka ihi oria gi (ukwu mgbu gi)?				Mba<br>Ee	1<br>2
D5.10	I ritere obere ego karia ka o di na mbu maka ihi oria gi (ukwu mgbu gi)?				Mba<br>Ee	1<br>2

If any of D5.5-D5.8 are rated greater than none (coded as "1"), ask:

D5.02	Na ubochi iri ato gara aga, ubochi ole ka I rughi oru okara ubochi ma o bu karia maka ihi oria gi (ukwu mgbu gi)?	***Record number of days.........***

Domain 6: Participation

Ugbu a, aga m aju gi ajuju gbasara etu isi esonye na ihe gbasara obodo gi na ka onodu ukwu mgbu gi si emetuta gi na ezi n'ulo gi. Ufodu ajuju a nwere ike igbasa nsogbu ndi i gabigara ubochi iri ato, mana ka I na aza ajuju ndi a, biko gbado anya na ubochi iri ato gara aga. Ozo, ana m echetara gi ka I zaa ajuju ndi a ka I na-eche banyere oria gi (ukwu mgbu gi).

Show flashcards #1 and #2

Ka mgbe abali iri ato gara aga:		Onweghi/O dighi	obere	ogafeghi oke	o siri ike	ogafere oke/enweghim ike ime
D6.1	Kedu oke nsogbu i nwere na iso ihe di iche iche a na eme na obodo gi (dika mmemme ndi obodo, mmemme ulo uka ma o bu ihe ndi ozo di iche iche) dika etu onye o bula nwere ike ime?	1	2	3	4	5
D6.2	Kedu oke nsogbu ole I nwere maka mgbochi ma o bu odachi na uwa gbara gi gburu gburu?	1	2	3	4	5
D6.3	Kedu oke nsogbu i nwere na ibi na ugwu diri mmadu maka ihi etu ndi mmadu si akpaso gi agwa na etu ha si emeso gi omume?	1	2	3	4	5
D6.4	Oge ole ka itinyere na oria gi (ukwu mgbu gi) ma o bu ihe si na ya puta?	1	2	3	4	5
D6.5	Kedu oke uzo esi metuta nmuo ma o bu mkpuru obi gi maka ihi oria gi (ukwu mgbu gi)?	1	2	3	4	5
D6.6	Kedu oke oria gi (ukwu mgbu gi) si erida ma o bu metuta onodu ego gi ma o bu ego ndi ezi n'ulo gi?	1	2	3	4	5
D6.7	Kedu oke nsogbu ezi n'ulo gi nwere maka ihi oria gi (ukwu mgbu gi)?	1	2	3	4	5

D6.8	Kedu oke nsogbu i nwere na iji aka gi eme otutu ihe ga enye gi ezumike ma o bu obi uto?	1	2	3	4	5

H1	Iji chikota ihe nile, na ubochi iri ato gara aga, na ime ubochi ole ka nsogbu ndi a biara?	***Record number of days..........***
H2	Na ubochi iri ato gara aga, na ime ubochi ole ka I na enweghi ike kpata kpata ime ihe I na adi eme na mbu ma o bu oru gi maka ihi oria gi (ukwu mgbu gi)?	***Record number of days..........***
H3	Na ubochi iri ato gara aga, na agunyeghi ubochi ndi I na enweghi ike kpata kpata, ubochi ole ka I wedatara aka na ihe ndi I na eme na mbu ma o bu oru maka oria gi (ukwu mgbu gi)?	***Record number of days..........***

Nke a bu njedebe ajuju onu a. Ndewo maka isonye.

***WHODAS FLASHCARD 1**

Health condition:

- Azu ukwu mgbu

Having difficulty with an activity means:

- I gbawanye mbo
- Mmekpa ahu ma o bu ihe mgbu
- Ime ihe nwayo
- Mgbanwe na uzo isi eme ihe

Think about the past 30 days only.

***WHODAS FLASHCARD 2**

1 **2** **3** **4** **5**

Onweghi /Odighi obere ogafeghi oke o siri ike ogafere oke/enweghi m ike ime
