# Supplementary material for: World Health Organisation Disability Assessment Schedule (WHODAS 2.0): development and validation of the Nigerian Igbo version in patients with chronic low back pain
Source: BMC Musculoskelet Disord. 2020 Nov 17;21:755. doi: 10.1186/s12891-020-03763-8 (PMC7670680; doi:10.1186/s12891-020-03763-8)
Supplement: Supplementary file 2 — Additional file 2. Changes made to Igbo-WHODAS following cross-cultural adaptation. [file 12891_2020_3763_MOESM2_ESM.pdf]

**Supplemental file 2: Alterations made to the Igbo World Health Organisation Disability Assessment Schedule (Igbo-WHODAS 2.0) following cross-cultural adaptation**

| | |
|---|---|
| **Instruction (translated exactly as in the original English version)** | In the past 30 days, how much difficulty did you have in: |

| **Item no** | **Original WHODAS 2.0** | **Exact English equivalent of the Igbo-WHODAS** |
|---|---|---|
| Items with 'how much' | How much | To what extent |
| D1.3 | Analysing and finding solutions to problems in day-to-day life? | Probing/exploring/researching and finding out/discovering solutions? |
| D4.3 | Getting along with people who are close to you? | Getting along/relating well with family and friends? |
| D6.1 | How much of a problem did you have joining in community activities (for example, festivities, religious or other activities) in the same way as anyone else can? | What is the extent of the problem you had in joining in activities that are performed in your community (such as community festivities, church festivities, and other festivities) in the same way anyone else can? |
| D6.5 | How much have you been emotionally affected by your health condition? | To what extent has your heart or spirit been affected by your health problem (your waist pain)? |
| D6.6 | How much has your health been a drain on the financial resources of you or your family? | To what extent has your illness (waist pain) been depleting or affecting the state of your finances or that of your family? |
