# Supplementary material for: World Health Organisation Disability Assessment Schedule (WHODAS 2.0): development and validation of the Nigerian Igbo version in patients with chronic low back pain
Source: BMC Musculoskelet Disord. 2020 Nov 17;21:755. doi: 10.1186/s12891-020-03763-8 (PMC7670680; doi:10.1186/s12891-020-03763-8)
Supplement: Supplementary file 3 — Additional file 3:. Reliability of Igbo-WHODAS. [file 12891_2020_3763_MOESM3_ESM.pdf]

Supplemental file 3: reliability of Igbo-WHODAS

Igbo-WHODAS total score

Number of items: 36; Cronbach's alpha global score: 0.97; ICC (95% CI): 0.93 (0.88, 0.96)

Cronbach's alpha If Item Deleted

D1.1	**D1.2**	**D1.3**	**D1.4**	**D1.5**	**D1.6**	**D2.1**	**D2.2**
0.97	0.97	0.97	0.97	0.97	0.97	0.97	0.97
**D2.3**	**D2.4**	**D2.5**	**D3.1**	**D3.2**	**D3.3**	**D3.4**	D4.1
0.97	0.97	0.97	0.97	0.97	0.97	0.97	0.97
**D4.2**	**D4.3**	**D4.4**	**D4.5**	**D5.1**	**D5.2**	**D5.3**	**D5.4**
0.97	0.97	0.97	0.97	0.97	0.97	0.97	0.97
**D5.5**	**D5.6**	**D5.7**	**D5.8**	**D6.1**	**D6.2**	D6.3	**D6.4**
0.97	0.97	0.97	0.97	0.97	0.97	0.97	0.97
**D6.5**	**D6.6**	**D6.7**	**D6.8**				
0.97	0.97	0.97	0.97				

SEM: 5.05 MDC: 13.99

Igbo-WHODAS 2.0 (cognition)

Number of items: 6; Cronbach's alpha global score: 0.88; ICC (95% CI): 0.87 (0.77, 0.93)

Cronbach's alpha If Item Deleted

D1.1	**D1.2**	**D1.3**	**D1.4**	**D1.5**	**D1.6**
0.85	0.87	0.86	0.87	0.86	0.88

SEM: 7.20 MDC: 19.96

Igbo-WHODAS 2.0 (mobility)

Number of items: 5; Cronbach's alpha global score: 0.91; ICC (95% CI): 0.90 (0.83, 0.94)

Cronbach's alpha If Item Deleted

D2.1	**D2.2**	**D2.3**	**D2.4**	**D2.5**
0.89	0.88	0.90	0.89	0.88

SEM: 8.00 MDC: 22.17

Igbo-WHODAS 2.0 (self-care)

Number of items: 4; Cronbach's alpha global score: 0.75; ICC (95% CI): 0.82 (0.68, 0.90)

Cronbach's alpha If Item Deleted

D3.1	**D3.2**	**D3.3**	**D3.4**
0.64	0.61	0.81	0.63

SEM: 7.20 MDC: 20.35

Igbo-WHODAS 2.0 (getting along with people)

Number of items: 5; Cronbach's alpha global score: 0.81; ICC (95% CI): 0.81 (0.66, 0.89)

Cronbach's alpha If Item Deleted

D4.1	**D4.2**	**D4.3**	**D4.4**	**D4.5**
0.76	0.76	0.74	0.76	0.83

SEM: 7.20 MDC: 20.35

Igbo-WHODAS 2.0 (life activities)

Number of items: 8; Cronbach's alpha global score: 0.95; ICC (95% CI): 0.93 (0.87, 0.96)

Cronbach's alpha If Item Deleted

D5.1	**D5.2**	**D5.3**	**D5.4**	**D5.5**	**D5.6**	**D5.7**	**D5.8**
0.94	0.94	0.94	0.94	0.94	0.94	0.94	0.94

SEM: 8.70 MDC: 24.11

Igbo-WHODAS 2.0 (participation)

Number of items: 8; Cronbach's alpha global score: 0.92; ICC (95% CI): 0.85 (0.73, 0.91)

Cronbach's alpha If Item Deleted

D6.1	**D6.2**	**D6.3**	**D6.4**	**D6.5**	**D6.6**	**D6.7**	**D6.8**
0.90	0.91	0.92	0.91	0.91	0.91	0.91	0.90

SEM: 11.10 MDC: 30.77
