# Supplementary material for: World Health Organisation Disability Assessment Schedule (WHODAS 2.0): development and validation of the Nigerian Igbo version in patients with chronic low back pain
Source: BMC Musculoskelet Disord. 2020 Nov 17;21:755. doi: 10.1186/s12891-020-03763-8 (PMC7670680; doi:10.1186/s12891-020-03763-8)
Supplement: Supplementary file 4 — Additional file 4. Bland-Altman plots of the Igbo-WHODAS. [file 12891_2020_3763_MOESM4_ESM.pdf]

Supplemental file 4: Bland-Altman plots of the Igbo-WHODAS

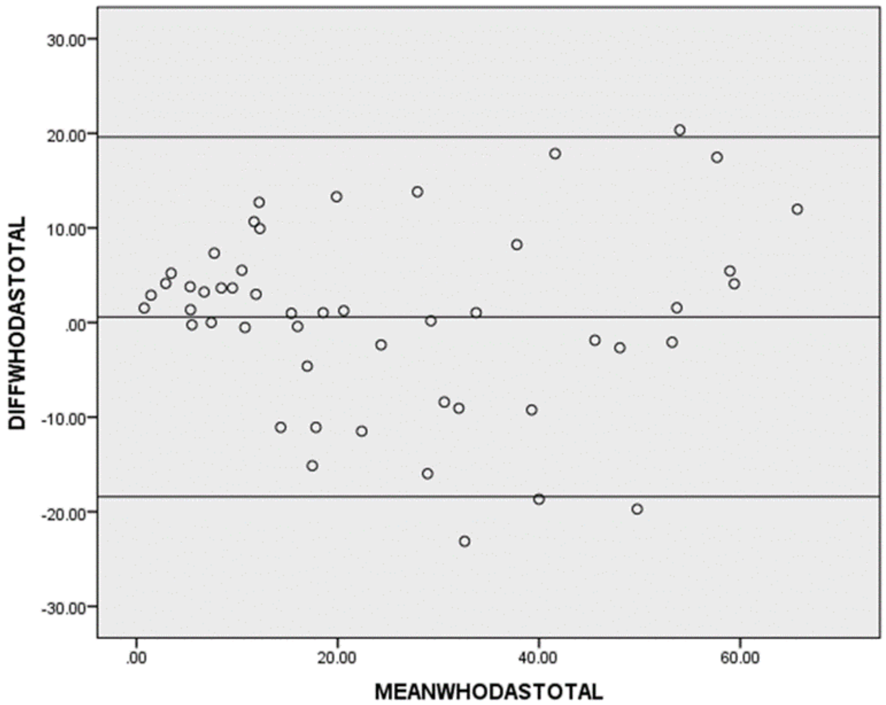

Figure 1: Bland-Altman plot for test-retest agreement of Igbo-WHODAS 2.0 (total score)

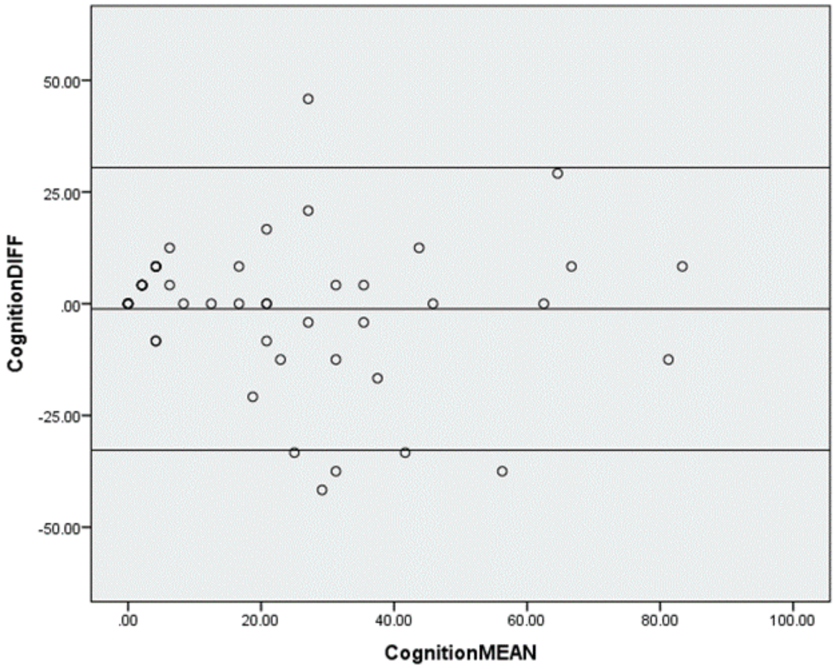

Figure 2: Bland-Altman plot for test-retest agreement of Igbo-WHODAS 2.0 (cognition)

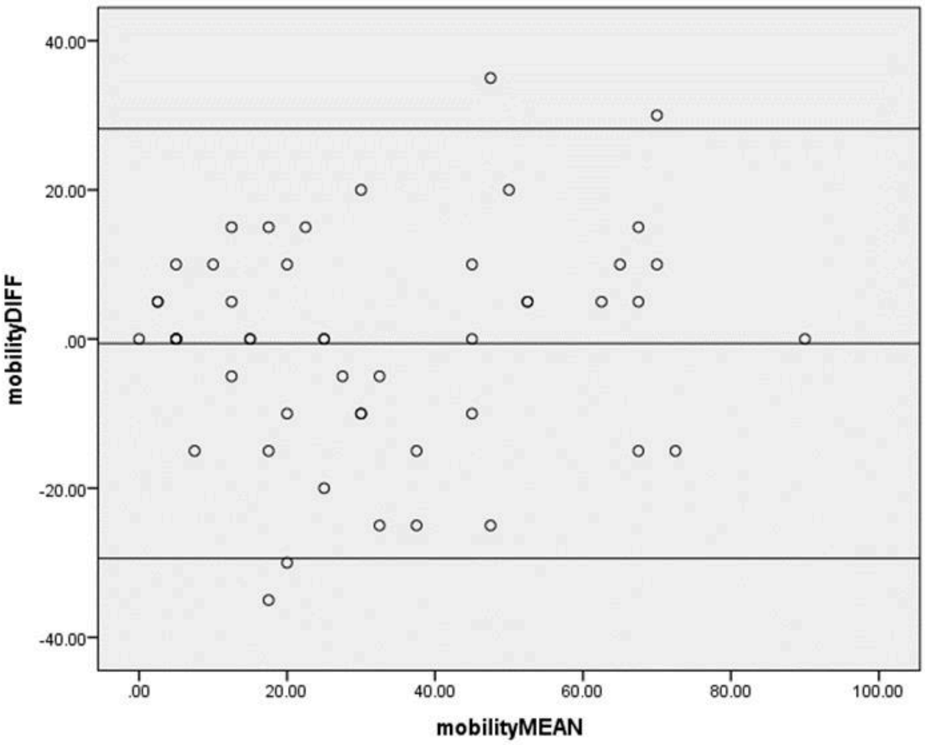

Figure 3: Bland-Altman plot for test-retest agreement of Igbo-WHODAS 2.0 (mobility)

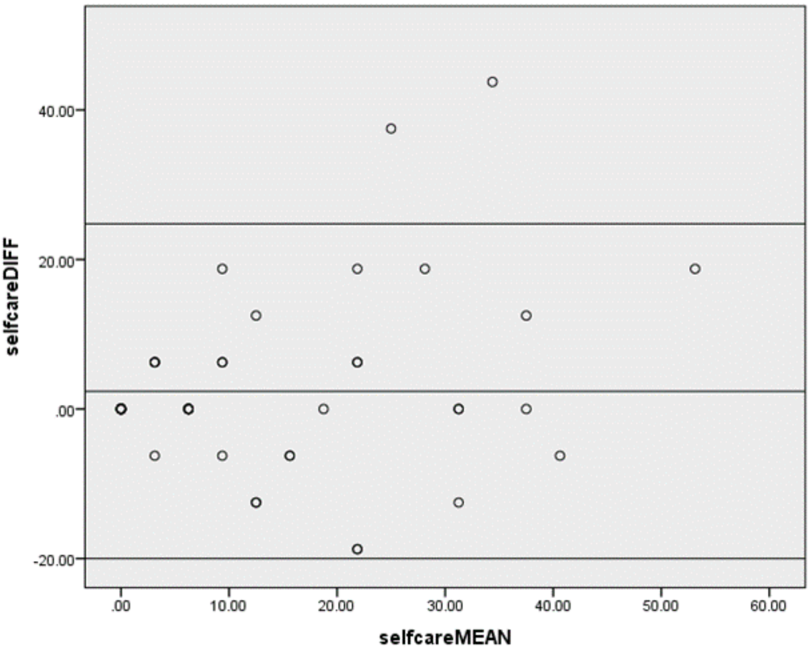

Figure 4: Bland-Altman plot for test-retest agreement of Igbo-WHODAS 2.0 (self-care)

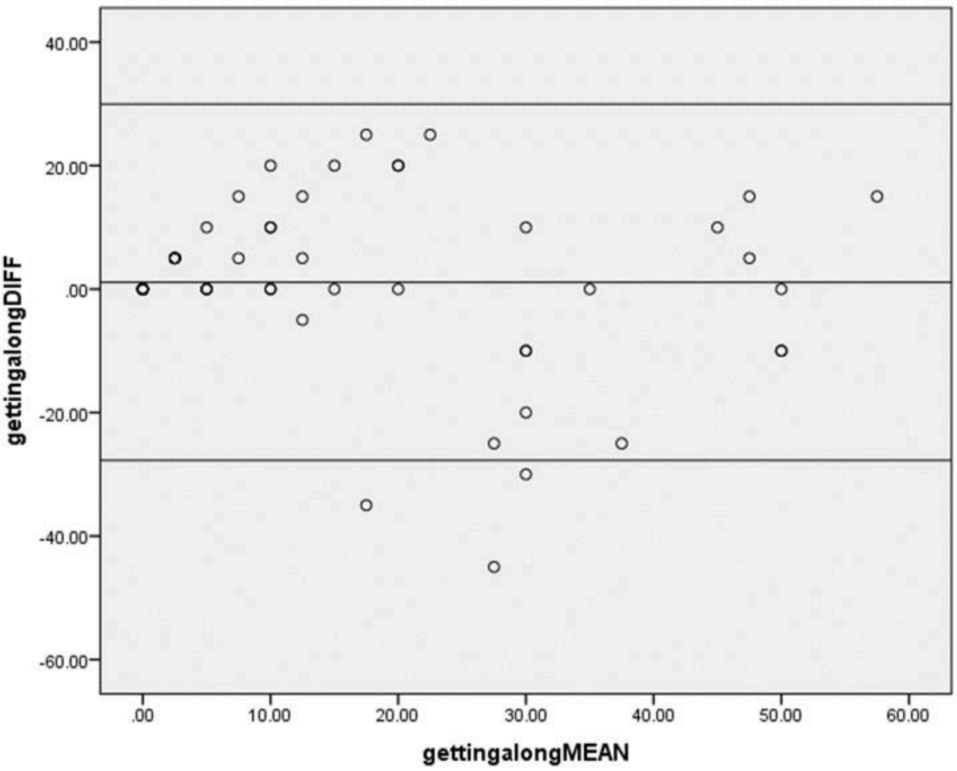

Figure 5: Bland-Altman plot for test-retest agreement of Igbo-WHODAS 2.0 (getting along with people)

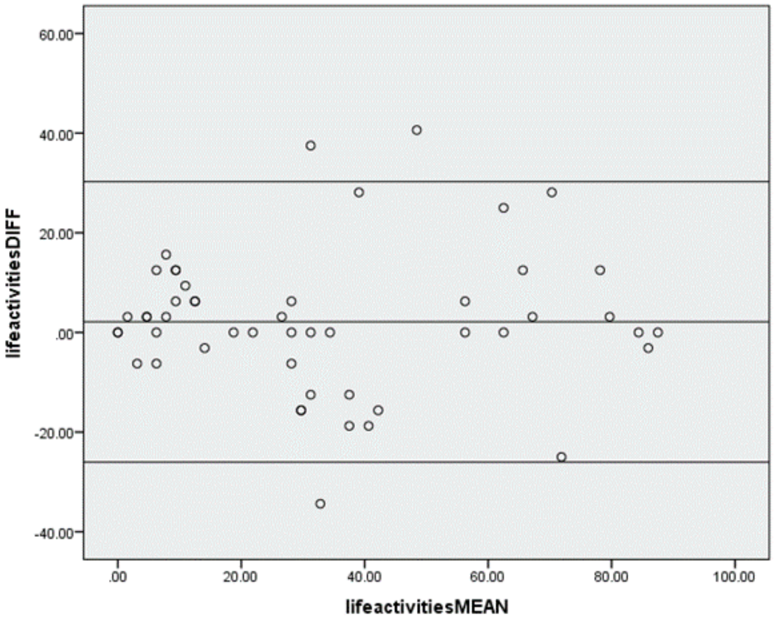

Figure 6: Bland-Altman plot for test-retest agreement of Igbo-WHODAS 2.0 (life activities)

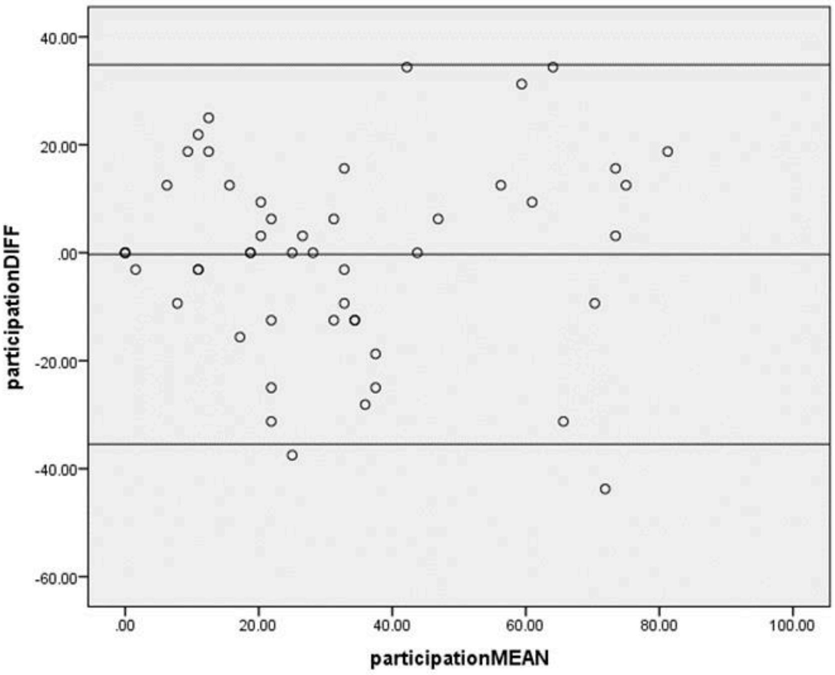

Figure 7: Bland-Altman plot for test-retest agreement of Igbo-WHODAS 2.0 (participation)
